# Testing small molecule analogues of the *Acanthocheilonema viteae* immunomodulator ES‐62 against clinically relevant allergens

**DOI:** 10.1111/pim.12322

**Published:** 2016-05-30

**Authors:** L. Janicova, J. Rzepecka, D. T. Rodgers, J. Doonan, K. S. Bell, F. E. Lumb, C. J. Suckling, M. M. Harnett, W. Harnett

**Affiliations:** ^1^ Strathclyde Institute of Pharmacy and Biomedical SciencesUniversity of StrathclydeGlasgowUK; ^2^ Institute of Infection, Immunity and InflammationUniversity of GlasgowGlasgowUK; ^3^ Department of Pure & Applied ChemistryUniversity of StrathclydeGlasgowUK

**Keywords:** allergen, asthma, ES‐62, parasitic worm, phosphorylcholine

## Abstract

ES‐62 is a glycoprotein secreted by the filarial nematode *Acanthocheilonema viteae* that protects against ovalbumin (OVA)‐induced airway hyper‐responsiveness in mice by virtue of covalently attached anti‐inflammatory phosphorylcholine (PC) residues. We have recently generated a library of small molecule analogues (SMAs) of ES‐62 based around its active PC moiety as a starting point in novel drug development for asthma and identified two compounds – termed 11a and 12b – that mirror ES‐62's protective effects. In this study, we have moved away from OVA, a model allergen, to test the SMAs against two clinically relevant allergens – house dust mite (HDM) and cockroach allergen (CR) extract. We show that both SMAs offer some protection against development of lung allergic responses to CR, in particular reducing eosinophil infiltration, whereas only SMA 12b is effective in protecting against eosinophil‐dependent HDM‐induced allergy. These data therefore suggest that helminth molecule‐induced protection against model allergens may not necessarily translate to clinically relevant allergens. Nevertheless, in this study, we have managed to demonstrate that it is possible to produce synthetic drug‐like molecules based on a parasitic worm product that show therapeutic potential with respect to asthma resulting from known triggers in humans.

## Introduction

During the past decade, there has been increasing interest in the idea that parasitic worms could be employed as novel treatments to control allergic diseases such as asthma. Although the idea originally arose from observations on humans, the most convincing supportive data come from studies with mouse models. Thus, infection with a wide range of different nematode and trematode species has been shown to protect mice against allergic lung disease (reviewed in 1, 2, 3). Furthermore, successful protection has also been obtained when employing individual defined parasitic worm products (reviewed in 1, 4). One of these defined products is ES‐62, a phosphorylcholine (PC)‐containing glycoprotein secreted by *Acanthocheilonema viteae*, which was shown to protect against disease development in a prophylactic version of the ovalbumin‐induced airway hypersensitivity model 5, 6. Furthermore, drug‐like small molecule analogues (SMAs) based around ES‐62's active PC moiety were found to be protective in this model and also when applied therapeutically 7. However, considering ovalbumin is a protein used to model physiologically relevant allergens, we have investigated whether these SMAs are also protective when administered to mice given allergens to which humans are naturally exposed, namely house dust mite (HDM) and cockroach (CR) extract.

## Materials and Methods

### Mice

Six‐ to eight‐week‐old C57BL/6 or BALB/c female mice were obtained from Harlan Olac (UK) or the Biological Procedures Unit (BPU), University of Strathclyde, UK. All animals were maintained in the BPU at the University of Strathclyde and housed in a pathogen‐free environment. All procedures were conducted in accordance with Home Office, UK animal guidelines (project licence number PPL60/4300) and with the approval of the local university ethical committees.

### HDM and CR extract models of allergic inflammation

**Figure 1:**
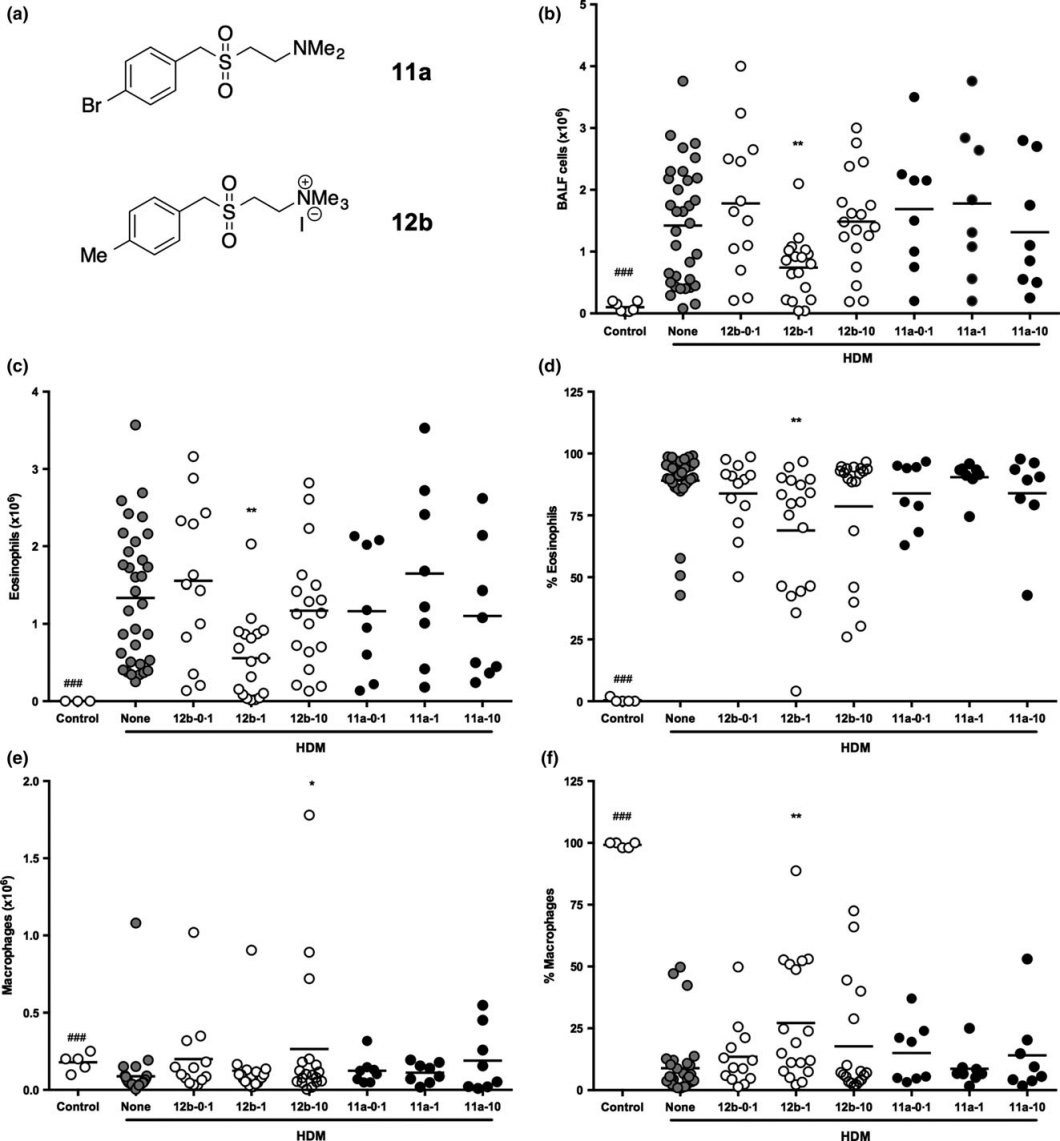
SMA 12b decreases total cell counts and influx of eosinophils into the lungs of mice undergoing HDM‐induced airway inflammation. C57BL/6 mice undergoing HDM‐induced airway inflammation were treated with SMA 11a or 12b 1 h before administering HDM extracts throughout the model. The numbers after each SMA refer to the quantity of μg of SMA per injection. (a) structures of SMAs 11a and 12b. (b) total cell count in BALF collected at sacrifice. Influx of eosinophils and macrophages is shown as both total number of cells (c, e) and their proportion (d, f) in BALF, as determined by differential cell counts. Data are combined from 5 independent experiments. Symbols on each graph are representative of individual mice in the indicated groups and where *P <* *0·05 and **P <* *0·01 for 12b‐1‐versus HDM (none) mice and ###*P *<* *0·001 for control vs. HDM mice.

Allergic inflammation was induced as previously described 8, 9. Briefly, mice were anaesthetized with isoflurane prior to intranasal (i.n.) administration of HDM (*Dermatophagoides pteronyssinus*) or CR (*Blattella germanica)* extract (Greer Laboratories, Lenoir, NC, USA), in sterile PBS to induce sensitization. For HDM‐induced allergic disease, mice were sensitized with 1 μg of HDM and challenged on days 7–11 by i.n. treatment with 10 μg doses of HDM extracts and the experiment was terminated on Day 14. For CR‐induced allergic disease, mice were sensitized with 50 μg of CR on days 0–4 and then challenged with 50 μg of CR on days 10–13 with termination of the model at Day 15. Control mice received 25 μL of PBS. The models are summarized in Figure S1. Endotoxin‐free SMA 11a and SMA 12b were prepared as described previously 10, and their structures can be seen in Figure 1a. Animals were anaesthetized using isoflurane and were treated with SMA 11a and SMA 12b (10, 1 or 0·1 μg/dose) either i.n. 1 h prior to HDM administration or subcutaneously (s.c) following CR administration where indicated. Disease‐positive control mice received vector treatment in place of SMAs, and untreated control mice received no SMA or HDM/CR. Serum, bronchoalveolar lavage (BALF), draining lymph nodes (DLNs) and lungs were removed, and *ex vivo* qRT‐PCR, ELISA, differential cell counts and flow cytometric analysis performed, as described previously 5, 6, 7. Flow cytometric analysis of cell populations utilized the following antibodies and fluorochromes: anti‐CD3ε/PerCP, anti‐Ly6C/FITC, anti‐Ly6G/APC, anti‐CD11c/PE‐Cy7 (all BioLegend, San Diego, CA, USA), anti‐CD19/APC‐Cy7, anti‐SiglecF/PE (BD Bioscience, San Diego, CA, USA) and anti‐F4/80/PE (eBioscience, San Diego, CA, USA).

### Lung histopathology

The tissue morphology was preserved by embedding lungs in 10% formalin solution for 24 h. Lungs were then embedded in Tissue‐Tek^®^ O.C.T.^™^ compound in Cryomolds and stored at −70°C until further analyses. Tissue sections (7 μm) were cut using a cryostat (Thermo Fisher, Renfrew, UK) and left to air‐dry for 24 h before haematoxylin and eosin (H&E) and periodic acid‐Schiff (PAS) staining were performed.

### Quantitative real time PCR (qRT‐PCR)

qRT‐PCR was undertaken as described previously 6 and using instructions provided in assay kits by Thermo Fisher. Briefly, the upper lobe from the right lung of the mouse was homogenized and used to extract RNA using an RNeasy Plus Mini Kit (Qiagen, Manchester, UK). The High Capacity cDNA Reverse Transcription Kit (Applied Biosystems, Paisley, UK) was used to reverse transcribe the obtained RNA, and the primers for IL‐4 (Mm00445259_m1), IL‐5 (Mm00439646_m1), IL‐13 (Mm00434204_m1), IL‐17 (Mm00439619_m1), IFN‐γ (Mm01168134_m1), IL‐1β (Mm00434228_m1), Hmox (Mm00516005_m1) and GAPDH (Mm99999915_g1) were used to analyse transcript levels. All samples were examined in duplicate, and data were analysed by steponeplus ^™^ software v 2.3 (Applied Biosystems, Paisley, UK) using the comparative *C*_T_ (ΔΔ*C*_T_) with values for samples being normalized to the reference reporter GAPDH.

### Detection of HDM/CR‐specific IgE and IgG1

ELISA 96‐well plates were coated with HDM or CR (2 μg/mL in PBS) overnight at 4°C and were then washed and blocked with 4% BSA/PBS (Sigma, Poole, Dorset, UK) for 1 h at 37°C. Serum was initially diluted 1/50 and then serially threefold 8 times prior to incubation in allergen‐coated plates at 37°C for 1 h. Horseradish peroxidase (HRP)‐conjugated anti‐IgE (anti‐mouse IgE‐HRP – 1 : 1000) and anti‐IgG1 (anti‐mouse IgG1‐HRP – 1 : 10 000) (antibodies from Southern Biotech, Birmingham, AL, USA) were then added and left for a further hour at 37°C to detect the presence of HDM/CR‐specific antibodies. Samples were developed by addition of KPL SureBlue, TMB microwell peroxidase substrate and reactions terminated by stop solution, 2N H_2_SO_4_. The final reactions were measured at 405 nm by employing an ELISA microplate reader.

### Lymphocyte restimulation assay

Single cell suspensions of lung mediastinal lymph nodes (3 × 10^5^ cells) were incubated with medium alone or CR (50 μg) for 72 h at 37°C. Cells were then pelleted at 400 × g, and supernatants collected for cytokine analysis. ELISAs were used to detect the concentration of IL‐4 and IFN‐γ according to the suppliers’ recommendations (BD Bioscience). Samples were developed using KPL SureBlue, TMB microwell peroxidase substrate and terminated by stop solution; 2N H_2_SO_4_ with the final reactions being measured at 405 nm using an ELISA microplate reader.

### Statistics

Data were transformed and analysed by one‐way anova using Bonferroni's (parametric) or Dunn's (nonparametric) *post hoc* tests.

## Results

### Effect of SMAs on cellular infiltration of the lungs in response to HDM extract

Two SMAs previously shown to be effective in preventing OVA‐induced inflammation in the lungs 7 were examined for their effects on cellular infiltration of the lungs in the HDM model.

To determine the optimum concentration of SMA to be employed, mice undergoing HDM‐induced airway hyper‐responsiveness were administered 11a or 12b, 1 h prior to HDM sensitization and at each challenge, at different concentrations – either 0·1 μg, 1 μg or 10 μg per injection – throughout the model (Figure S1). A control group (‘none’) received the DMSO vehicle instead of SMA. Mice were culled on Day 14, and bronchoalveolar lavage fluid (BALF) was collected for total and differential cell count analysis. SMA 11a did not show protective effects at any of the three concentrations employed (Figure 1b). Likewise, when SMA 12b was administered to mice at concentrations of 0·1 μg or 10 μg per injection, the total number of cells in BALF remained unaffected in such animals, when compared to the group of untreated mice undergoing HDM‐induced airway hyper‐responsiveness (Figure 1b). However, when 12b was administered at a concentration of 1 μg per injection, the mean total number of cells decreased significantly compared to untreated HDM mice (7·4 × 10^5^ vs. 1·4 × 10^6^ for total number of BALF cells; *P *<* *0·01; Figure 1b). The mean total cell count values for 12b administered at 0·1 μg and 10 μg per injection were 1·8 × 10^6^ and 1·5 × 10^6^, respectively. For comparison, mice not exposed to HDM (‘control’) had a mean total number of cells in the lungs of 1·0 × 10^5^. Eosinophils were not detected in the BALF of such mice, whilst a mean number of 1·3 × 10^6^ (89%) was detected in HDM‐treated mice (Figure 1c). However, this influx of eosinophils was reduced with the 1 μg SMA/injection regimen in a similar manner to the decrease in total cells (Figure 1c,d). Indeed, whilst at the low and high concentrations of 12b employed, the total numbers of eosinophils were similar to those detected in the untreated HDM group (1·6 × 10^6^ (84%) and 1·2 × 10^6^ (79%), respectively; Figure 1b), at the effective middle concentration of 1 μg of 12b per injection, this number was significantly decreased to 5·6 × 10^5^ (69%; *P *<* *0·01). Consistent with the total cell data (Figure 1b), SMA 11a had no effect on the HDM‐induced eosinophil influx (Figure 1c,d).

By contrast to the eosinophil data, the absolute numbers of macrophages were found to increase in the BALF of mice given SMA 12b at 10 μg per injection (Figure 1e), whilst administration at 1 μg per injection induced an increase in their levels as a proportion of BALF cells that reached statistical significance when compared to the untreated HDM group, with the percentage increasing from 8·9 to 27·2 (Figure 1f). The mean proportion of macrophages remained comparable to the HDM group for 12b employed at 0·1 μg (13·5%) and 10 μg/injection (17·8%) groups. No changes in the number (Figure 1e) or proportion (Figure 1f) of macrophages were observed when administering SMA 11a. In spite of the observed percentage increase following administration of 12b at 1 μg, flow cytometric analysis of the myeloid cells in the lung tissue (results not shown) did not detect a significant difference in either the M1 or M2 macrophage subsets or the CD11b^+^ or CD103^+^ DC populations 11. The lymphocyte count was below 5 × 10^4^ cells and remained unchanged amongst the experimental groups, and neutrophils did not appear to be recruited into the lungs of mice in any of the groups (data not shown).

**Figure 2:**
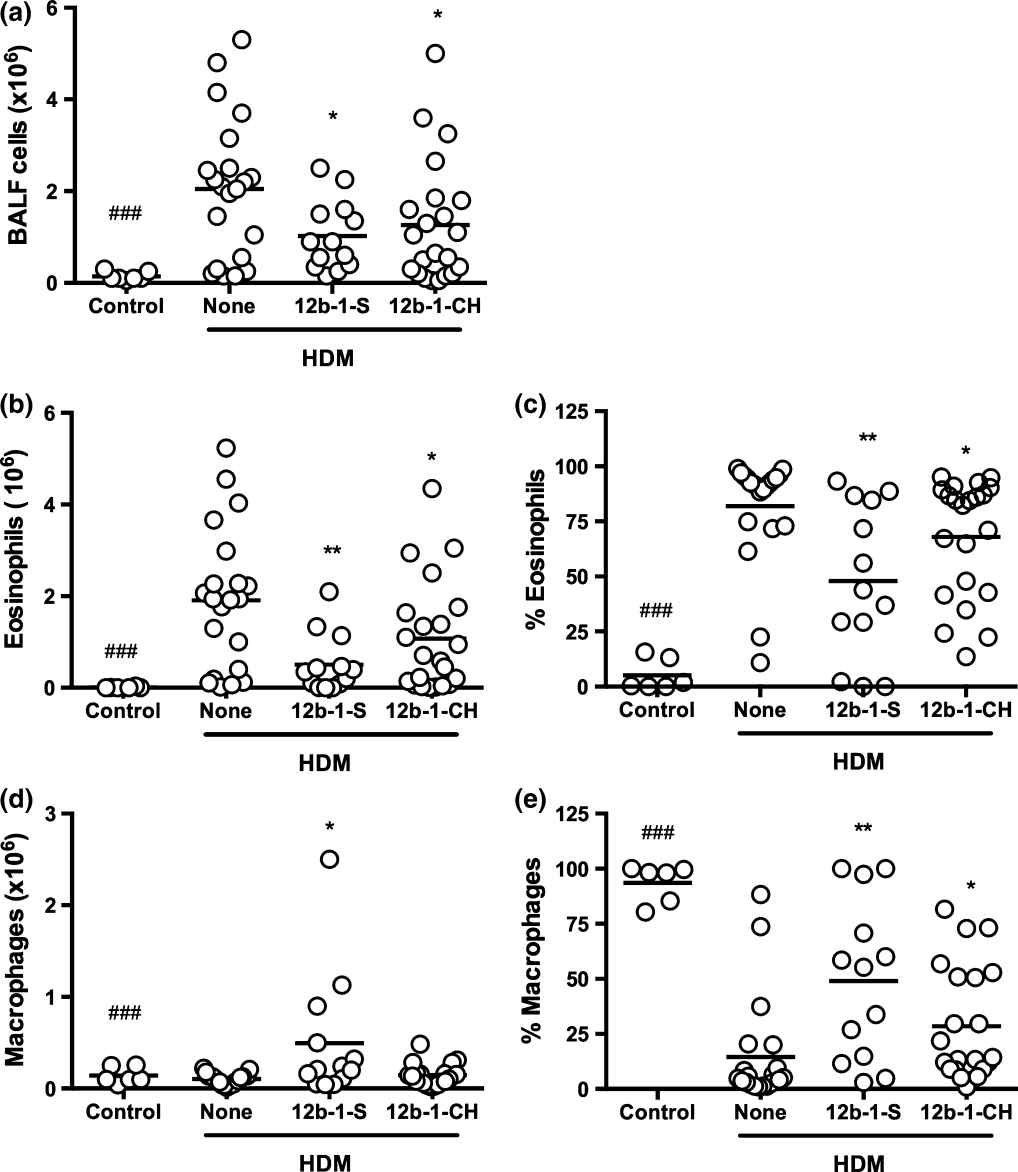
SMA 12b prevents influx of cells into the lungs of mice undergoing HDM‐induced airway inflammation when administered either prophylactically or therapeutically. C57BL/6 mice undergoing HDM‐induced airway hyper‐responsiveness were treated with SMA 12b 1 h before either sensitization (S) or challenges (CH) with HDM extracts. (a) total cell count in BALF collected at sacrifice. Influx of eosinophils and macrophages is shown as both total number of cells (b, d) and their proportion (c, e) in BALF. Data are pulled from 3 independent experiments. Symbols on each graph are representative of individual mice in the indicated groups and where *P <* *0·05 and **P <* *0·01 *P*‐value is indicated in comparison with the HDM (none) control and ###*P *<* *0·001 for control vs. HDM mice.

To determine the effectiveness of SMA 12b at its active concentration of 1 μg/injection at different time points in preventing the pathology associated with HDM‐induced airway hypersensitivity, the SMA was administered either prophylactically (at the sensitization stage only; 12b‐1‐S) or therapeutically (at the challenges only; 12b‐1‐CH). Total cell count in BALF of mice undergoing HDM‐induced airway hyper‐responsiveness showed that SMA 12b, when administered prophylactically, was able to suppress the influx of cells into the lungs of mice significantly compared to non‐SMA‐treated HDM mice (10·2 × 10^5^ vs. 2·0 × 10^6^ for total number of BALF cells; *P *<* *0·05; Figure 2a). This decrease was consistent with a decrease in eosinophils (from 1·9 × 10^6^ cells to 5·1 × 10^5^; *P *<* *0·01; Figure 2b). At the same time, an increase in total macrophage count was observed with SMA treatment (from 1·03 × 10^5^ cells to 4·9 × 10^5^; *P *<* *0·05; Figure 2d). Proportion wise, the data represented a decrease from 82% to 48% for eosinophils (*P *<* *0·05; Figure 2c) and increase from 14·5% to 49% of macrophages (*P *<* *0·05; Figure 2e).

Similarly, when SMA 12b was administered therapeutically (at the challenges only), there was a decrease in influx of cells into the lungs of HDM‐treated mice (from 1·9 × 10^6^ cells to 1·3 × 10^6^; Figure 2a), although this was less pronounced relative to prophylactic administration. Nevertheless, this slight decrease in total cells in BALF was consistent with a statistically significant decrease in total eosinophils (from 1·9 × 10^6^ cells to 1·1 × 10^6^; *P *<* *0·05; Figure 2b). Proportion wise, this represented a decrease from 82% to 68% (Figure 2c). Whilst no significant effects of the SMA were observed with respect to the number (Figure 2d), the proportion (Figure 2e) of macrophages was significantly increased from 14·5% to 28·5% when the SMA was only administered prior to HDM challenges.

### Effect of SMAs on lung pathology in the HDM model

**Figure 3:**
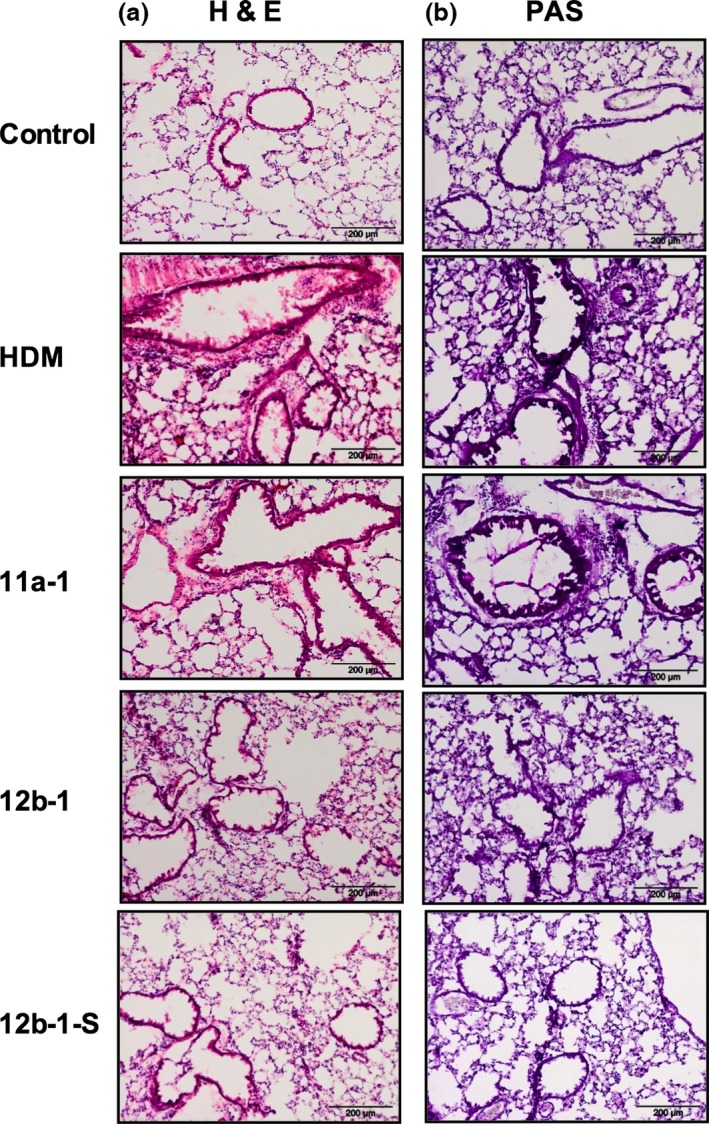
Histochemical staining of lung tissue from mice treated with SMA 11a or 12b at different time points. Alveolar regions of lung sections (7 μm) stained with H&E (a) and periodic acid‐Schiff (PAS; b) from C57BL/6 mice. Three different regions of lungs were examined in mice from two independent experiments, and representative figures are displayed. The control group was not exposed to any treatment. The HDM group represents mice exposed to HDM extracts in vehicle only. 11a‐1 and 12b‐1 represent groups of mice treated with either 11a or 12b at 1 μg per injection throughout the model. 12b‐1‐S represents a group of HDM mice treated with 12b prophylactically at the sensitization stage only. Sections were examined using an Olympus BX41 light microscope at ×40 magnification and scale bars represent 200 μm.

At the cull day, lungs were collected from mice for assessment of inflammation and pathology (Figure 3). 11a‐1 and 12b‐1 represent groups of mice treated with either 11a or 12b at 1 μg per injection throughout the model. 12b‐1‐S represents a group of HDM mice treated with 12b prophylactically at the sensitization stage only. H&E staining of mouse lung sections revealed histopathological changes in mice exposed to HDM extract. In particular, cellular infiltration and an increase in airway thickness, which occurs due to an upsurge in smooth muscle deposition around the airways, in combination with irregularity in the structure of the airways, were observed in the HDM‐treated mice, and this was similar to what was observed in the 11a‐1‐treated group. These changes were less obvious in the SMA 12b‐1 group, as was mucus production, indicated by way of PAS staining, within the airways. Also, when mice were treated with 12b throughout the model, airway remodelling seemed to be suppressed. Furthermore, shedding of cells was less visible resulting in more normal structures being observed in the airways. Similarly, when 12b was administered prophylactically at the sensitization only stage (12b‐1‐S group), lung pathology was reduced in all aspects, to a level comparable to the lungs of mice in the 12b‐1 group. Likewise, when 12b was administered just before challenge, we witnessed a reduction in the degree of lung inflammation (results not shown).

We also stained sections with toluidine blue in an attempt to detect mast cells as this cell type has been previously shown to be a target of both ES‐62 5 and SMAs 7. However, as discussed previously 7, it is not always possible to find significant numbers of mast cells in the lungs of mice employed in asthma models. Consistent with this, we could not detect increased numbers in HDM‐treated mice as compared to control mice (results not shown).

### Effect of SMAs on cytokine gene expression in the HDM model

**Figure 4:**
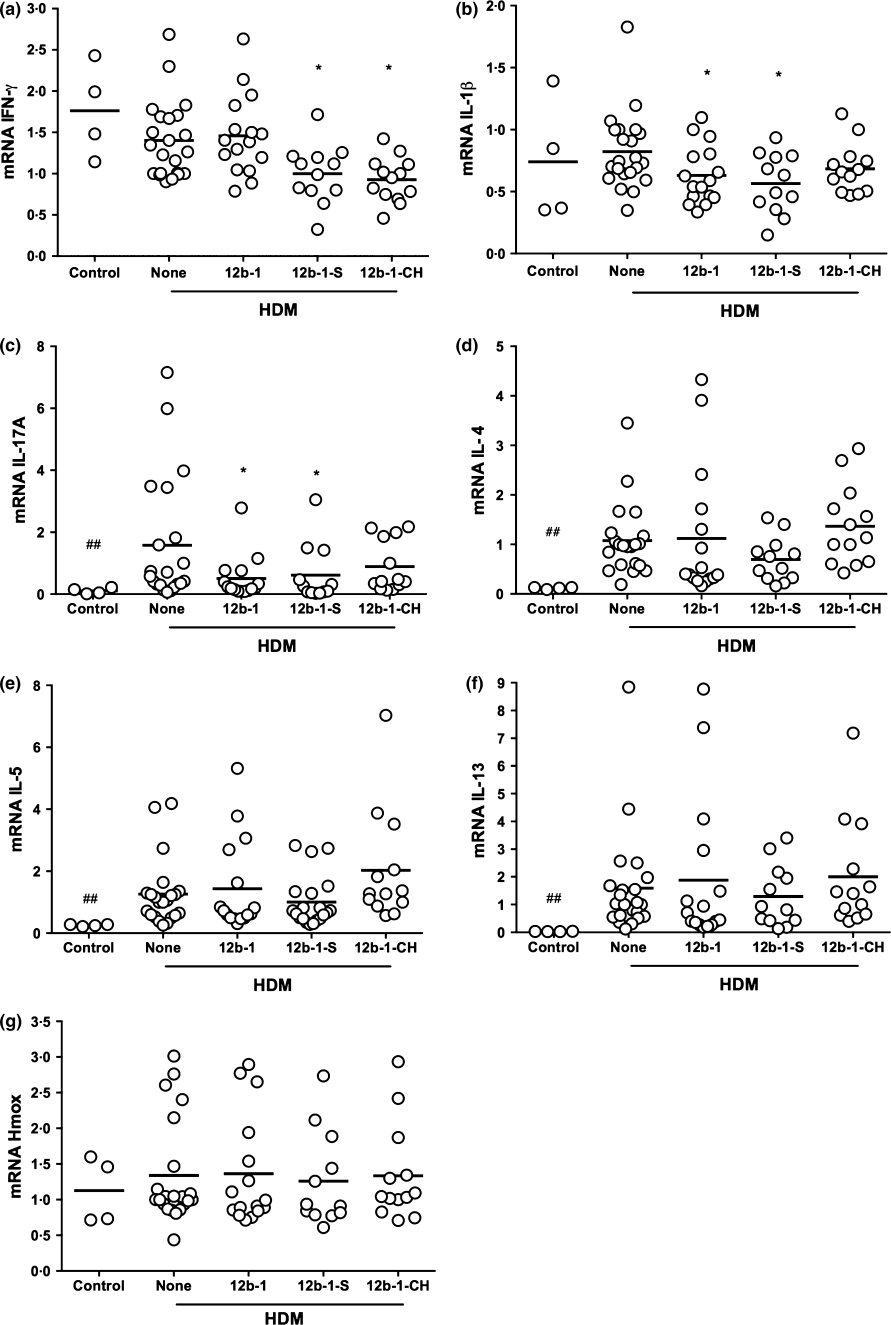
Effect of ES‐62 SMA 12b on cytokine production in the lungs as measured by qRT‐PCR. qRT‐PCR analysis of IFN‐γ (a), IL‐1β (b), IL‐17A (c), IL‐4 (d), IL‐5 (e), IL‐13 (f) and Hmox (g) mRNA levels in the lungs of HDM mice treated with 12b throughout the model (12b‐1), either prophylactically at the sensitization only (12b‐1‐S) or therapeutically at the challenges only (12b‐1‐CH) stage. The data are pooled from three independent experiments with values for samples being normalized to the reference reporter for GAPDH. Each symbol represents the response from an individual mouse in the designated group and where *P <* *0·05 indicates comparison between HDM (none) and HDM‐SMA‐treated mice and ##*P *<* *0·01 for control vs. HDM mice.

To elucidate the mechanism of action of SMA 12b in the HDM model of asthma, expression of a number of proinflammatory cytokines, in particular, IFN‐γ, IL‐1β, IL‐17A, IL‐4, IL‐5 and IL‐13, was examined. In addition, the levels of Heme Oxygenase‐1 (Hmox) that produces protective antioxidants 12 were analysed as imbalance in these factors is commonly implicated in asthma 13. As Figure 4 shows, the lungs of mice exposed to HDM demonstrated a statistically significant increase in expression of IL‐17A, IL‐4, IL‐5 and IL‐13 relative to untreated mice (all *P *<* *0·01). No significant effects were observed with respect to the other cytokines examined or Hmox in the lungs. When measuring expression of these molecules in the DLNs, only IL‐5 and IL‐13 were significantly increased by HDM treatment (both *P *<* *0·05; Figure S2). Perhaps, consistent with this Th17/Th2 polarization, HDM treatment tended to reduce IFN‐γ mRNA levels in the lungs (Figure 4a). Treatment with SMA 12b throughout significantly lowered the increased levels of IL‐17A mRNA in the lungs of mice undergoing airway hypersensitivity (Figure 4c), and although the slight rise in IL‐1β mRNA levels resulting from HDM treatment did not reach significance, a significant decrease was measured in the lungs for the group of mice receiving 12b‐1 (Figure 4b). These were the only significant reversals of HDM‐induced effects attributable to treatment with 12b‐1 throughout the experiment in either the lungs (Figure 4) or DLN (Figure S2). In addition, and consistent with its lack of effect on HDM pathology, treatment with SMA 11a at 1 μg/injection throughout the model did not significantly modulate the mRNA levels of any of the mediators tested in lungs (Figure S3) and DLNs (Figure S4) as compared to HDM‐treated mice.

Levels of IL‐1β and IL‐17A mRNA were also decreased in the lungs for the group treated with 12b only at sensitization, whereas these effects were not apparent for the group of mice receiving 12b therapeutically at the HDM challenges (Figure 4b,c). Like HDM treatment alone, 12b‐1 also tended to reduce the levels of IFN‐γ mRNA relative to those pertaining in the control untreated mice and this was also found for both 12b‐1‐S and 12b‐1‐CH where the decreases reached statistical significance (Figure 4a). No effects of 12b‐1‐S or 12b‐1‐CH treatments were detected for any of the mediators in the DLNs at cull (Figure S2).

### Effect of SMAs on the IgG1 antibody response in the HDM model

**Figure 5:**
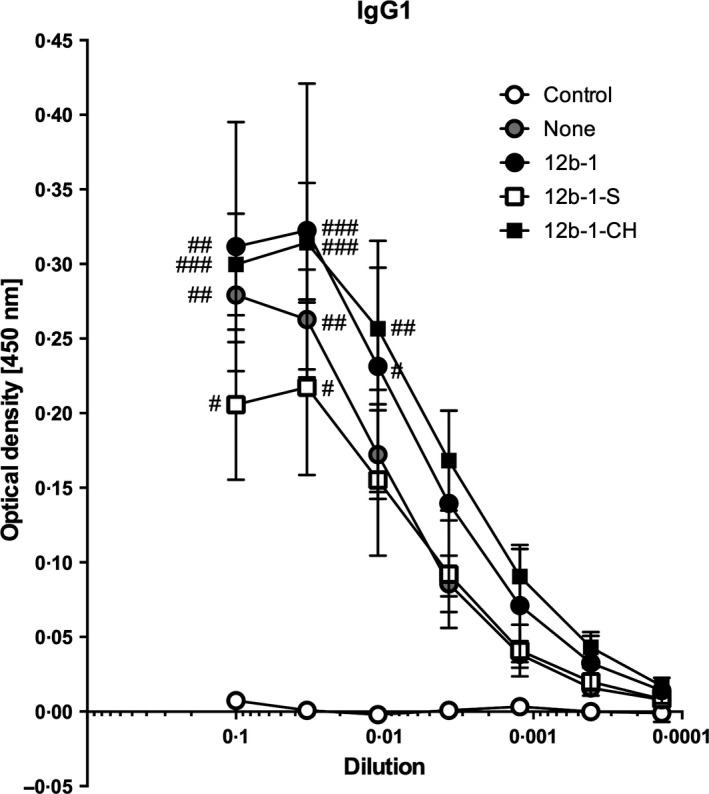
Measurement of HDM‐specific IgG_1_ levels following treatment with SMA 12b. Serum from HDM‐hypersensitive mice treated with 12b at different time points was obtained at the cull day. Serum samples were diluted threefold, serially and concentrations for HDM‐specific IgG_1_ levels measured by ELISA. The symbols on the graph show mean values, and SEM of individual mouse IgG_1_ levels collated from two independent experiments. #*P* < 0*·*05; ##*P* < 0*·*01; ###*P* < 0*·*001 *vs*. control (no HDM) at each dilution point.

To further investigate the protective effects of SMA 12b, the levels of HDM‐specific IgG1 immunoglobulins were assessed. It was found however that there was no difference in specific antibody levels when mice hypersensitive to HDM extracts received treatment with 12b‐1 at any of the time points – at the sensitization, throughout the model or at the challenges only in comparison with mice receiving HDM extract alone (Figure 5). Consistent with this, flow cytometric analysis revealed that exposure to 12b‐1 did not modulate the levels or phenotype of effector (Marginal Zone, Follicular, Germinal Centre, Plasmablast or Plasma Cell) or putative regulatory IL‐10^+^ B cells 14, 15 in the spleen (data not shown).

### Testing of SMAs in the cockroach (CR) extract allergen model

**Figure 6:**
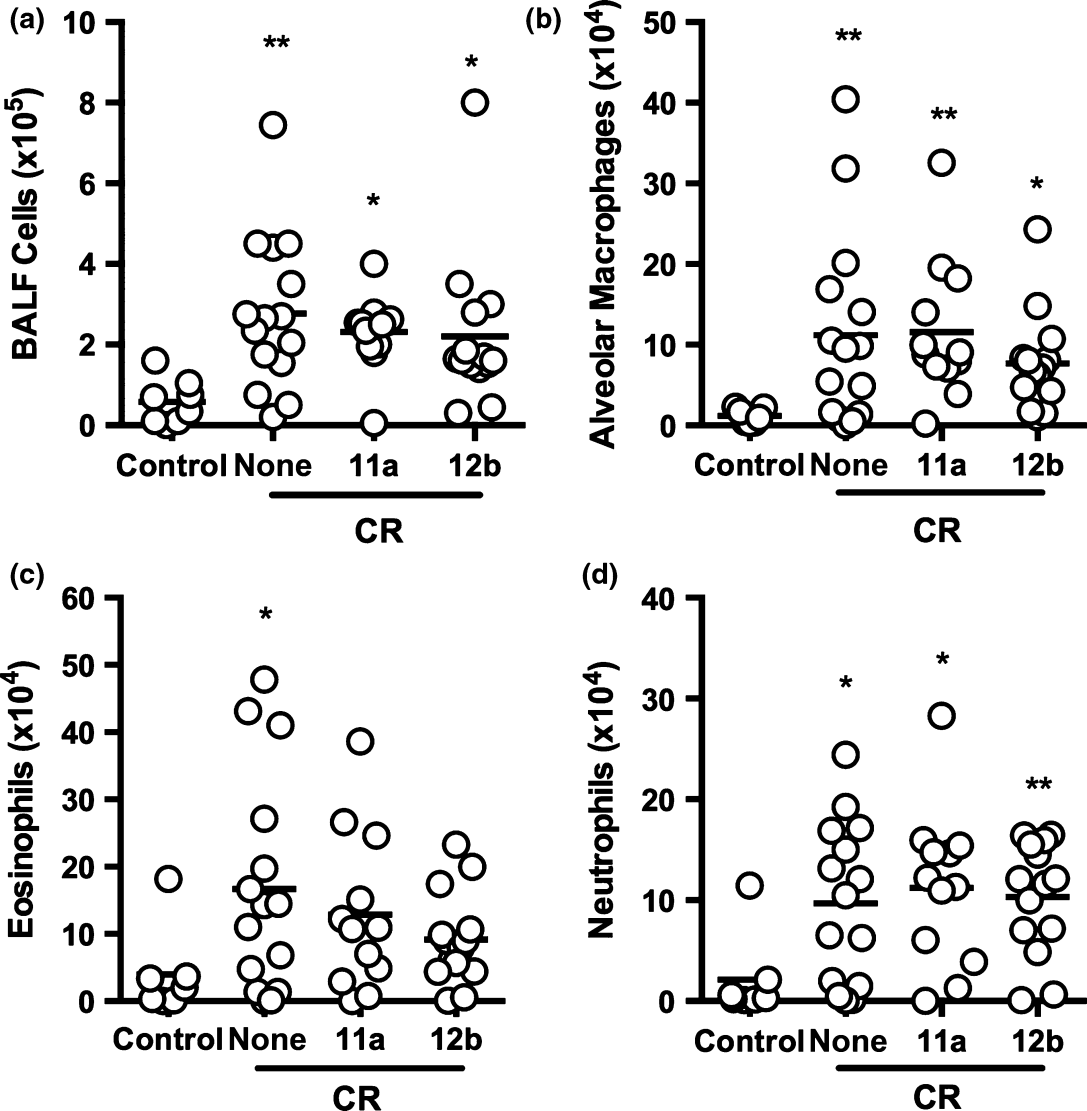
SMAs 11a and 12b inhibit influx of eosinophils into the lungs of mice undergoing CR‐induced airway inflammation. BALB/c mice undergoing CR‐induced airway hyper‐responsiveness (‘none’) were treated with SMAs 11a or 12b 1 h prior to each exposure to CR. (a) total cell count in BALF collected at sacrifice. Influx of alveolar macrophages (b: Ly6C^‐^Ly6G^‐^ CD3^‐^ CD19^‐^ CD11c^+^F4/80^+^), eosinophils (c: SSC ^high^SiglecF^+^) and neutrophils (d: SSC ^high^Ly6G^+^) is shown as total number of cells in BALF. Data are pooled from 3 independent experiments. Symbols on each graph are representative of individual mice in the indicated groups. One of the control mice was identified as an outlier by the GraphPad Prism ROUT test in all of these cell types and hence was excluded from the statistical analyses where *P <* *0·05 and **P <* *0·01 and the *P*‐value is indicated in comparison with the control (no CR exposure) mice.

**Figure 7:**
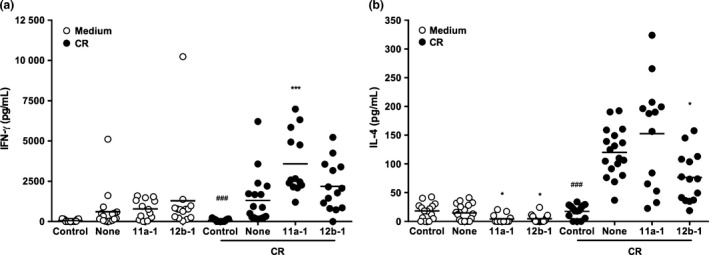
Effect of ES‐62 SMAs on lymphocyte cytokine production. DLN cells (3 × 10^5^ lymphocytes) were taken from control, CR‐ or CR‐SMA‐treated animals and cultured *ex vivo* with medium alone or CR (50 μg) for 72 h. Cell culture supernatants were removed, and IFN‐γ (a) and IL‐4 levels measured by ELISA. The data presented are pooled from 3 independent experiments and *P <* *0·05 and ***P <* *0·001 are for untreated CR‐exposed mice (none) vs. SMA‐treated CR‐exposed mice and ###*P* < 0·001 for control (no CR exposure) vs. CR‐exposed (none) mice.

Mice were exposed to CR (15 or 50 μg/injection) during sensitization, and challenge (Figure S1) and total and differential cell counts in BALF determined. This analysis revealed that exposure to CR at 50 μg per injection resulted in significantly higher levels in the BALF of total cells and also alveolar macrophages, eosinophils, neutrophils and lymphocytes (Figure 6 and results not shown; see Figure S5 for flow cytometric gating strategy). Whilst exposure to SMAs 11a and 12b employed at 1 μg per injection did not have any significant effects on total or alveolar macrophage, neutrophil or lymphocyte cell counts found in mice treated with CR extract, the levels of eosinophils found in the BALF of such treated animals were not significantly different to those found in naive untreated mice, indicating the SMAs were acting to suppress eosinophilic infiltration of the airways in this model (Figure 6 and results not shown). A statistically significant IgG1 antibody response was observed in 1 of 3 experiments, but SMAs 11a and 12b had no effect on this (results not shown). However, when measuring cytokine responses in DLNs, 11a significantly increased the IFN‐γ (Figure 7a) and 12b inhibited the IL‐4 (Figure 7b) recall response to CR.

## Discussion

We have previously reported that ES‐62 and its drug‐like SMAs 11a and 12b provide protection against OVA‐induced airway hyper‐responsiveness in a mouse model 5, 6, 7. Testing of helminths, their extracts or their defined products on allergic airway inflammation has become increasingly common over the past decade (reviewed in 1, 2, 4) with the vast majority of the studies focusing on OVA. Nevertheless, there are a few studies, which rather than employ this model allergen, focus on molecules that induce allergy in humans such as Derp1 from HDM 16 and grass pollen allergen 17. The present work extends these studies, employing both an HDM and a CR extract to induce allergic airway responses in the lungs of mice.

SMAs 11a and 12b were initially tested in the HDM model using three different doses of SMA per injection – 0·1, 1 and 10 μg – administered 1 h prior to each exposure to HDM throughout the model. 11a had no effect on the HDM‐induced increase in cells in the BALF, but 12b was effective in significantly reducing this when employed at 1 μg/injection. The reason underlying 12b only being efficacious at the mid‐dose level is unknown: we did not observe such an effect in the OVA model 7 although we have seen some evidence of it in collagen‐induced arthritis (CIA) where a log increase in the quantity of SMA beyond 1 μg/injection reduces the protective effect (Pineda, M.A., Harnett, M.M. & Harnett, W., unpublished data). Parabolic dose–responses may not be unusual 18 as such concentration/activity profiles are possible where a system under investigation is not responding to a single‐target/single‐drug intervention, which would typically exhibit a simple concentration dependence. In this case, we could argue, for example, that SMA 12b has a high affinity target that is reflected in the initial concentration‐dependent increase of activity observed. However, if it also has a secondary low affinity target that signals the reverse of the initial activity, this effect would be seen at higher concentration. Alternatively, and perhaps more consistent with our findings that ES‐62/SMAs act to reset homeostatic regulatory mechanisms, at higher concentration that saturate responses from the high affinity target, there could be compensating mechanisms within the cell that act to restore the original state. This would obviate the need for the SMA acting at a second lower affinity target. Three concentration points are, of course, not enough to define a profile but could be argued to be indicative of interacting signalling mechanisms in the cells. Clearly, more pharmacological analysis would have to be undertaken to help resolve this issue. Likewise, the reason for the failure of 11a to protect in the model is uncertain as such a result was not observed in the OVA model 7. Nevertheless, although the structures of 11a and 12b are based upon the same conceptual design 10, it is already apparent that they have somewhat different properties *in vivo*. For example, although the two SMAs offer protection against pathology in a model of oxazolone‐induced skin inflammation 19 and the MRL/Lpr model of systemic lupus erythematosus 20 in both cases, SMA 11a is the more effective. Thus, we may simply be observing differences in the efficacy of the SMAs commensurate with differences in their structures (see Figure 1 for structures) the importance of which needs to be unravelled in relation to the different models. Of note, SMA 12b was also found to be effective when administered only before sensitization or only before challenge indicating that it has both prophylactic and therapeutic potential with respect to employment as a drug.

Differential cell analysis revealed that SMA 12b targeted eosinophil infiltration, and this was the case regardless of when the SMA was administered. Previously, we had shown that 12b inhibited eosinophil infiltration in the prophylactic version of the OVA model, but the neutrophil was found to be the target cell in a therapeutic model 7. However, no neutrophil infiltration of the lungs was observed in the present study, and hence, anti‐neutrophil effects could not be measured. Further analysis revealed that 12b but not 11a had additional effects on the airways of animals undergoing HDM‐induced allergy including reducing mucus production and preventing airway thickening and remodelling. Such observations increase the likelihood that 12b may have both prophylactic and therapeutic potential as a treatment for allergic airway disease, even after the onset of lung pathology associated with chronic asthma and hence may have application to each of mild, moderate and severe asthma (the last being particularly difficult to treat). In addition, 12b (and 11a) is a prototype compound suitable for proof‐of‐concept experiments such as these: the structures have not as yet been optimized and so it can be anticipated that both potency and selectivity can be improved in development.

Induction of allergy in the lungs via HDM resulted in an increase in mRNA for IL‐4, IL‐5, IL‐13 and IL‐17. Although 12b when administered throughout the model had no effect on the Th2 cytokines (the lack of effect on IL‐4 may also help explain the lack of effect on specific IgG1 antibody levels), it reduced expression of IL‐17, a cytokine reported as contributing to eosinophil activation 21. Previously, we have observed that both ES‐62 6 and 12b 7 reduce IL‐17 mRNA levels in the lungs when employing the prophylactic OVA model although on this occasion, IL‐4 was also inhibited (the other Th2 cytokines were not measured). The cell type responsible for the IL‐17 production that is being targeted by 12b in the HDM model remains to be established, but it is known from our studies on CIA that the parent molecule ES‐62 can inhibit IL‐17 production in a number of ways including direct and indirect (via APC) effects on IL‐17‐producing cells such as Th17 cells and γδ T cells 22. Of note, 12b does not appear to directly target IL‐17 responses in CIA, focusing instead on IL‐1β 23. However, consistent with this latter result, although not significantly altered by HDM‐induced allergy, IL‐1β mRNA levels are reduced by 12b treatment in this model. IFN‐γ levels are dampened in a similar manner although these tend to be unaltered by 12b 7 and indeed elevated by ES‐62 6, in the OVA model. Thus, the protective effects associated with reversal of Th polarity observed previously in the OVA model 6, 7 do not appear to pertain here. In the HDM model, effects on converging IL‐1β and IL‐17 signalling in lung pathology may thus be more important. Certainly, the latter cytokine has been reported to play a key role in the HDM model 24, which makes the failure of SMA 11a – an SMA known to target IL‐17 responses 7, 10 – somewhat surprising.

Regarding the effect of the SMAs on other cell types known to be targets, as with neutrophils, we could not find increased numbers of mast cells in the lungs of HDM‐exposed mice, and hence, it appears that this cell type does not represent an obvious component of the inflammatory response in this model. Consistent with this, we did not find any increase in HDM‐specific serum IgE levels (results not shown). We also considered the B cell as a target because it has emerged from our work on mouse models of rheumatoid arthritis 15, systemic lupus erythematosus 14 and more recently a chronic OVA‐based asthma model 25 that protection afforded by ES‐62 involves resetting the homeostatic balance between regulatory (Breg) and effector B cells. Our findings relating to Bregs were disappointing however as we found 12b to only induce small insignificant increases in splenic IL‐10^+^ B cells in HDM mice at cull (d14, from about 1·5 to 1·7%). Nevertheless, a number of the parasitic worm studies have now demonstrated that the protective efficacy of such putative Breg populations depends on their localization (reviewed in 25) indicating that the spleen, although the organ traditionally investigated for the induction and function of such cells, may not have been the best site of investigation. Also of note, our data in the chronic OVA model have revealed the protective induction of MZ‐like Bregs by ES‐62 and 12b to be acute and dynamic raising the possibility that we may have missed the peak of any Breg induction. This may additionally suggest that if SMAs do induce Bregs in the HDM model, they are likely to be using this same acute mechanism of action at both sensitization and challenge. Certainly, this would be consistent with what appears to be a somewhat acute inhibition of inflammatory responses rather than a repolarization of Th responses, as would be reflected by modulation of the levels of cytokines, such as IL‐4. In any case, we plan to further explore these matters at a later date.

Finally, in contrast to the HDM model, both SMAs 11a and 12b exhibited some protective effects on CR‐induced allergic airway inflammation with both, as in the OVA model 7, tending to reduce eosinophilic infiltration of the lungs. Although neutrophilic infiltration was induced by CR, this was not modified by either 11a or 12b, unlike the situation in the OVA model where prophylactic exposure to 12b also inhibited this inflammatory response 7. However, we did observe effects on IFN‐γ (increased) and IL‐4 (decreased) DLN recall cytokine responses, which may be consistent with the suppression of eosinophil responses, in that IL‐4 is known to increase the number of this cell type in the airways and lungs of mice in an antigen‐dependent manner 26, as well as with the anti‐allergy effects noted with ES‐62 and the SMAs in the OVA model 6, 7. Also, when we then moved on to employ the CRE model, the published regimens most resembled our prophylactic OVA model where SMAs had been successfully administered subcutaneously 7. However, although evidence of protection has been obtained in both models in the present study, obviously, without exploring all routes of administration in all models, we cannot know which are most effective. Thus, further experimentation may be warranted here although this will probably await the development of second generation SMAs based around the structures of 11a and 12b. Certainly, overall, exploitation of the HDM and CR models in addition to the OVA model not only allows us to test ‘clinically relevant’ allergens, but increases our knowledge of the selectivity in activity of the SMAs in different inflammatory contexts; importantly, in the case of 11a, it has also shown that what works with a model allergen may not necessarily afford protection against all members of the ‘real thing’.

## Supporting information


**Figure S1.** Schematic of HDM and CR models of airway hyper‐responsiveness. For the HDM model, C57BL/6 animals were treated intranasally with SMAs 11a or 12b (10, 1 and 0·1 μg/dose) 1 h prior to administration of HDM extract (1 or 10 μg/dose). The cockroach extract model utilized BALB/c animals that were treated with SMAs 11a or 12b (1 μg/dose) subcutaneously prior to each intranasal administration of CR (15 or 50 μg/dose).

**Figure S2.** Effect of ES‐62 SMA 12b on cytokine production in the draining lymph nodes as measured by qRT‐PCR. qRT‐PCR analysis of IFN‐γ (a), IL‐1β (b), IL‐17A (c), IL‐4 (d), IL‐5 (e), IL‐13 (f) and Hmox (g) mRNA levels in the lungs of HDM mice treated with 12b either throughout the model (12b‐1), prophylactically at the sensitization only (12b‐1‐S) or therapeutically at the challenges only (12b‐1‐CH) stage. The data are pooled from three independent experiments with values for sample being normalized to the reference reporter for GAPDH. Each symbol represents the response from individual mice in the designated group and where ##*P* < 0·05 for untreated (“Control”) vs. HDM‐treated (“None”) mice.

**Figure S3.** Effect of ES‐62 SMA 11a on cytokine production in the lungs as measured by qRT‐PCR**.** qRT‐PCR analysis of IFN‐γ (a), IL‐1β (b), IL‐17A (c), IL‐4 (d), IL‐5 (e), IL‐13 (f) and Hmox (g) mRNA levels in the lungs of HDM mice untreated (“None”) or treated with 1 μg injections of 11a (“11a‐1”) throughout the model. The data are from a single experiment with values for samples being normalized to the reference reporter for GAPDH. Each symbol represents the response from individual mice in the designated group.

**Figure S4.** Effect of ES‐62 SMA 11a on cytokine production in the draining lymph nodes as measured by qRT‐PCR. qRT‐PCR analysis of IFN‐γ (a), IL‐1β (b), IL‐17A (c), IL‐4 (d), IL‐5 (e), IL‐13 (f) and Hmox (g) mRNA levels in the lungs of HDM mice untreated (“None”) or treated with 1 μg injections of 11a (“11a‐1”) throughout the model. The data are from a single experiment with values for samples being normalized to the reference reporter for GAPDH. Each symbol represents the response from individual mice in the designated group.

**Figure S5.** Representative flow cytometric gating strategy for the CR extract model. For the flow cytometric phenotypic analysis, the cells of interest and exclusion of doublets were determined by the forward and side scatter parameters of the cellular populations (a, b). Siglec F was used to identify Eosinophils (c: SSC^high^SiglecF^+^), while a separate staining panel was used to identify Neutrophils (d: SSC^high^Ly6G^+^). The Ly6G^‐^ population in D was further discriminated on the basis of their Ly6C expression (e) and following selection of the Ly6C^‐^Ly6G^−^ CD3^−^CD19^−^ cells, the subsequent use of F4/80 and CD11c allowed the identification of Alveolar Macrophages (f: Ly6C^−^Ly6G^−^CD3^−^CD19^−^CD11c^+^F4/80^+^).
