# Supplementary figures and images for: Testing small molecule analogues of the Acanthocheilonema viteae immunomodulator ES‐62 against clinically relevant allergens
Source: Parasite Immunol. 2016 May 30;38(6):340–51. doi: 10.1111/pim.12322 (PMC4913752; doi:10.1111/pim.12322)

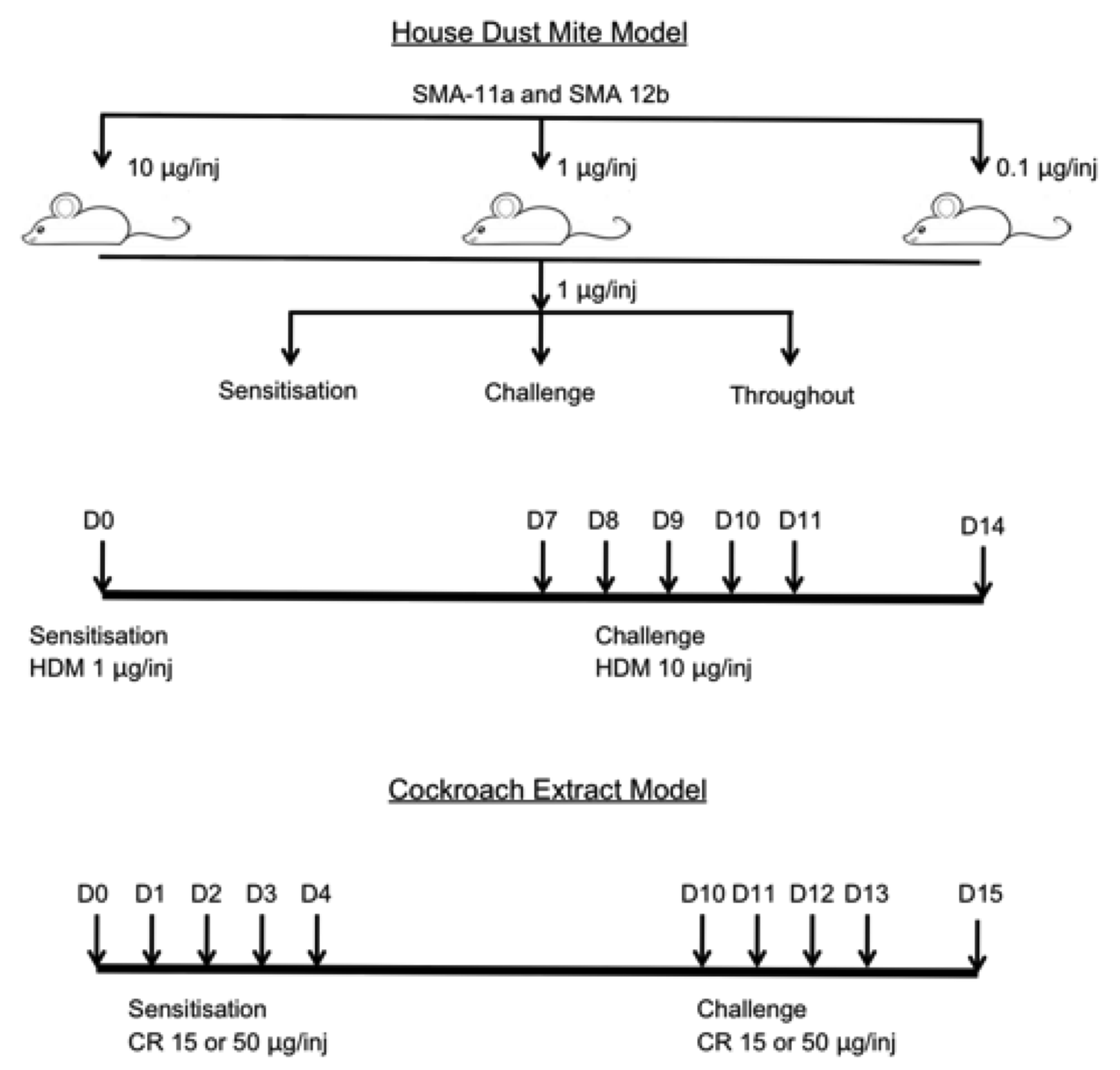

Supplement: Supplementary file 1 — Figure S1. Schematic of HDM and CR models of airway hyper‐responsiveness. For the HDM model, C57BL/6 animals were treated intranasally with SMAs 11a or 12b (10, 1 and 0·1 μg/dose) 1 h prior to administration of HDM extract (1 or 10 μg/dose). The cockroach extract model utilized BALB/c animals that were treated with SMAs 11a or 12b (1 μg/dose) subcutaneously prior to each intranasal administration of CR (15 or 50 μg/dose). [file PIM-38-340-s001.tiff]

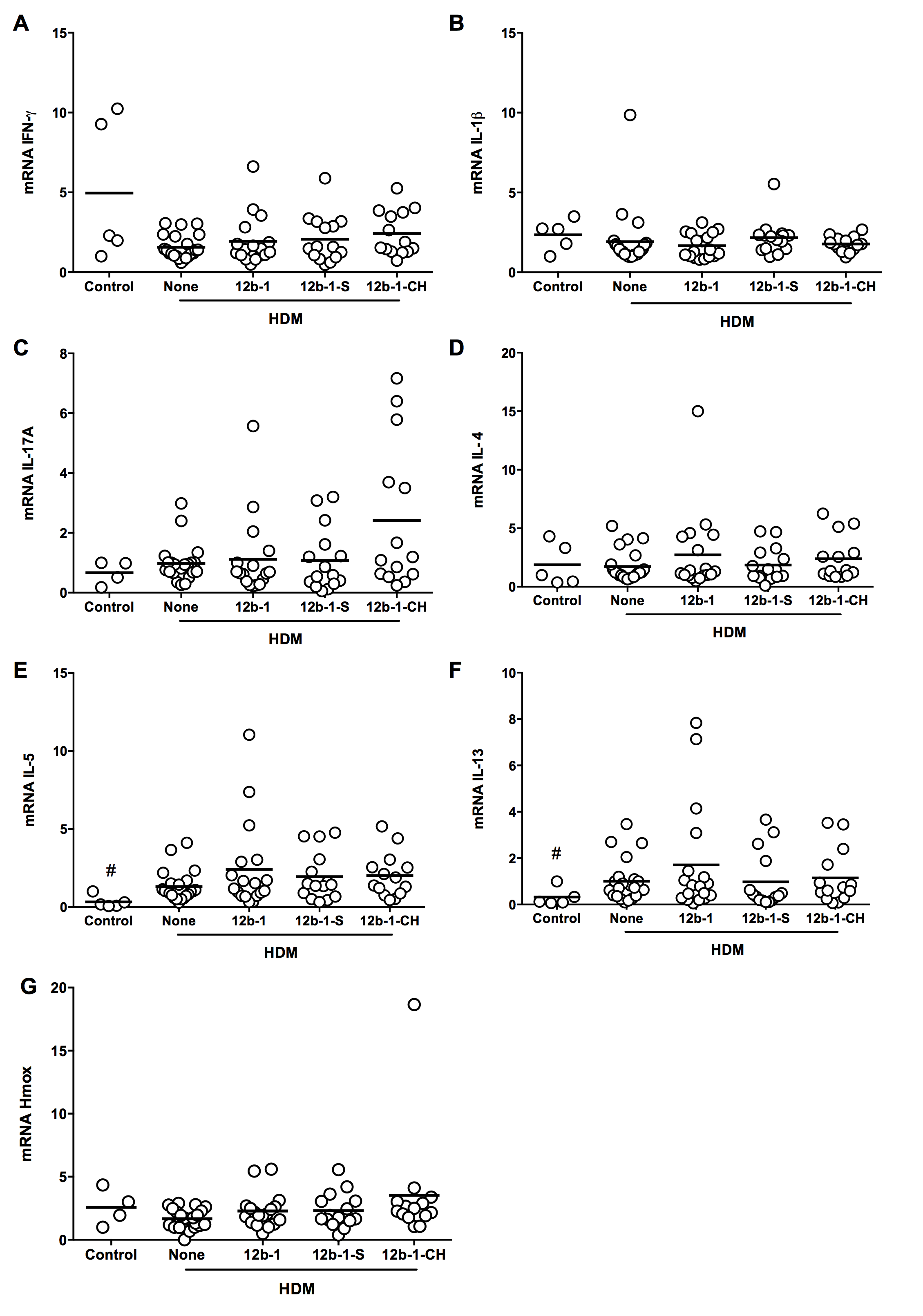

Supplement: Supplementary file 2 — Figure S2. Effect of ES‐62 SMA 12b on cytokine production in the draining lymph nodes as measured by qRT‐PCR. qRT‐PCR analysis of IFN‐γ (a), IL‐1β (b), IL‐17A (c), IL‐4 (d), IL‐5 (e), IL‐13 (f) and Hmox (g) mRNA levels in the lungs of HDM mice treated with 12b either throughout the model (12b‐1), prophylactically at the sensitization only (12b‐1‐S) or therapeutically at the challenges only (12b‐1‐CH) stage. The data are pooled from three independent experiments with values for sample being normalized to the reference reporter for GAPDH. Each symbol represents the response from individual mice in the designated group and where ##P < 0·05 for untreated (“Control”) vs. HDM‐treated (“None”) mice. [file PIM-38-340-s002.tiff]

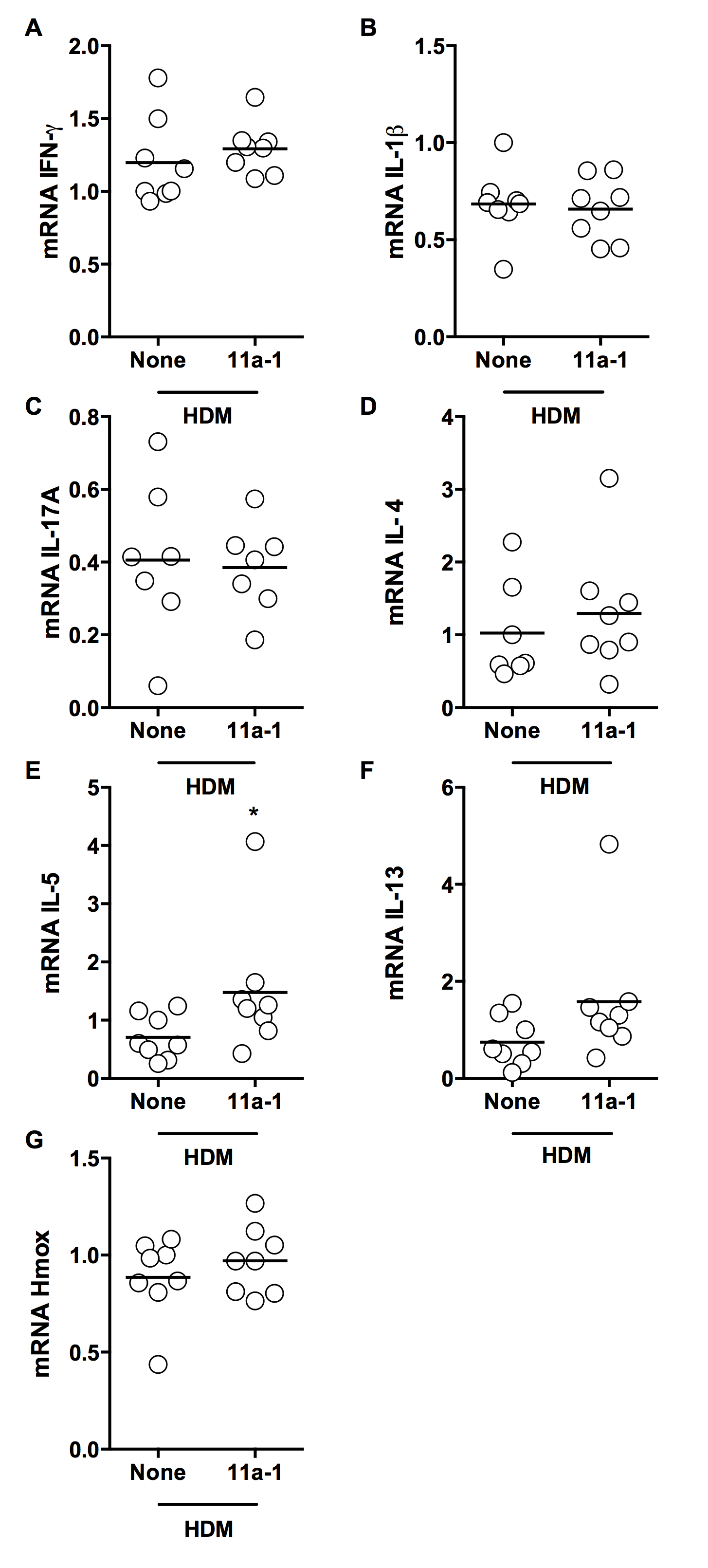

Supplement: Supplementary file 3 — Figure S3. Effect of ES‐62 SMA 11a on cytokine production in the lungs as measured by qRT‐PCR. qRT‐PCR analysis of IFN‐γ (a), IL‐1β (b), IL‐17A (c), IL‐4 (d), IL‐5 (e), IL‐13 (f) and Hmox (g) mRNA levels in the lungs of HDM mice untreated (“None”) or treated with 1 μg injections of 11a (“11a‐1”) throughout the model. The data are from a single experiment with values for samples being normalized to the reference reporter for GAPDH. Each symbol represents the response from individual mice in the designated group. [file PIM-38-340-s003.tiff]

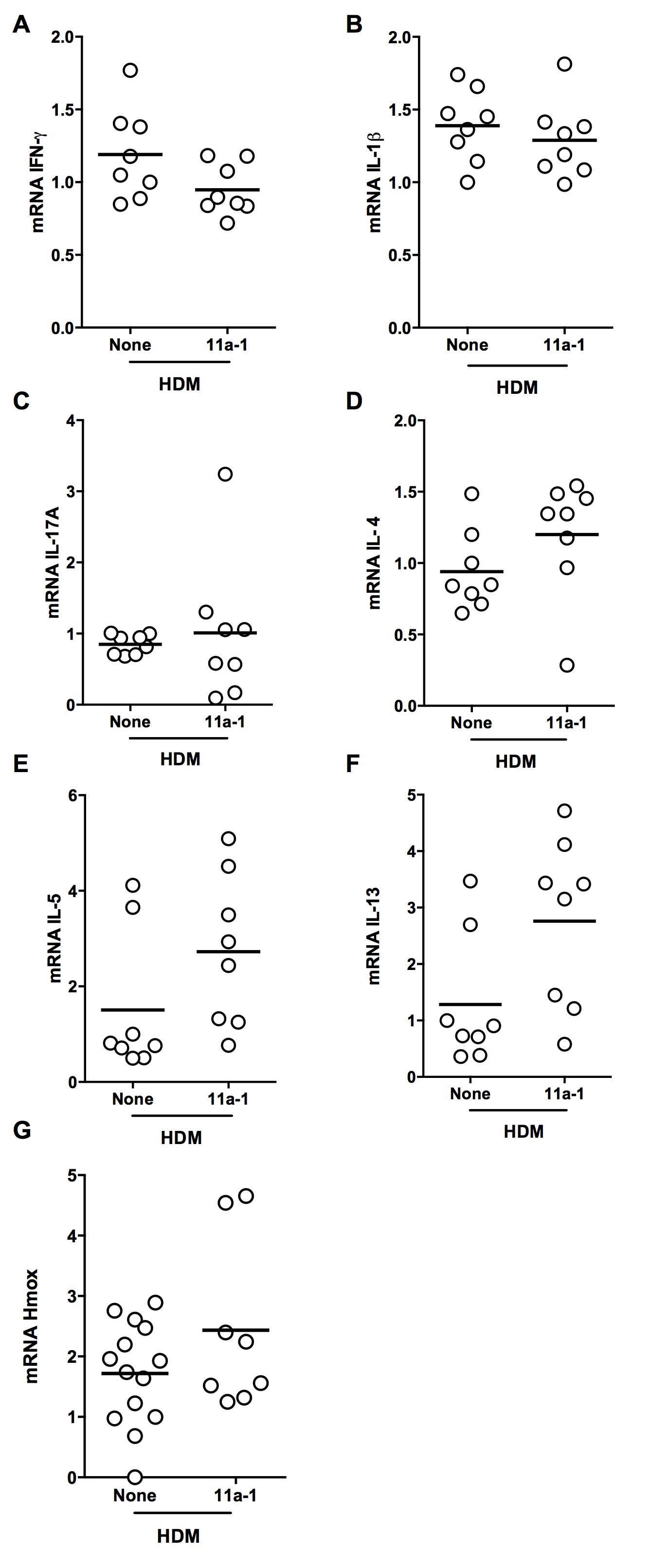

Supplement: Supplementary file 4 — Figure S4. Effect of ES‐62 SMA 11a on cytokine production in the draining lymph nodes as measured by qRT‐PCR. qRT‐PCR analysis of IFN‐γ (a), IL‐1β (b), IL‐17A (c), IL‐4 (d), IL‐5 (e), IL‐13 (f) and Hmox (g) mRNA levels in the lungs of HDM mice untreated (“None”) or treated with 1 μg injections of 11a (“11a‐1”) throughout the model. The data are from a single experiment with values for samples being normalized to the reference reporter for GAPDH. Each symbol represents the response from individual mice in the designated group. [file PIM-38-340-s004.tiff]

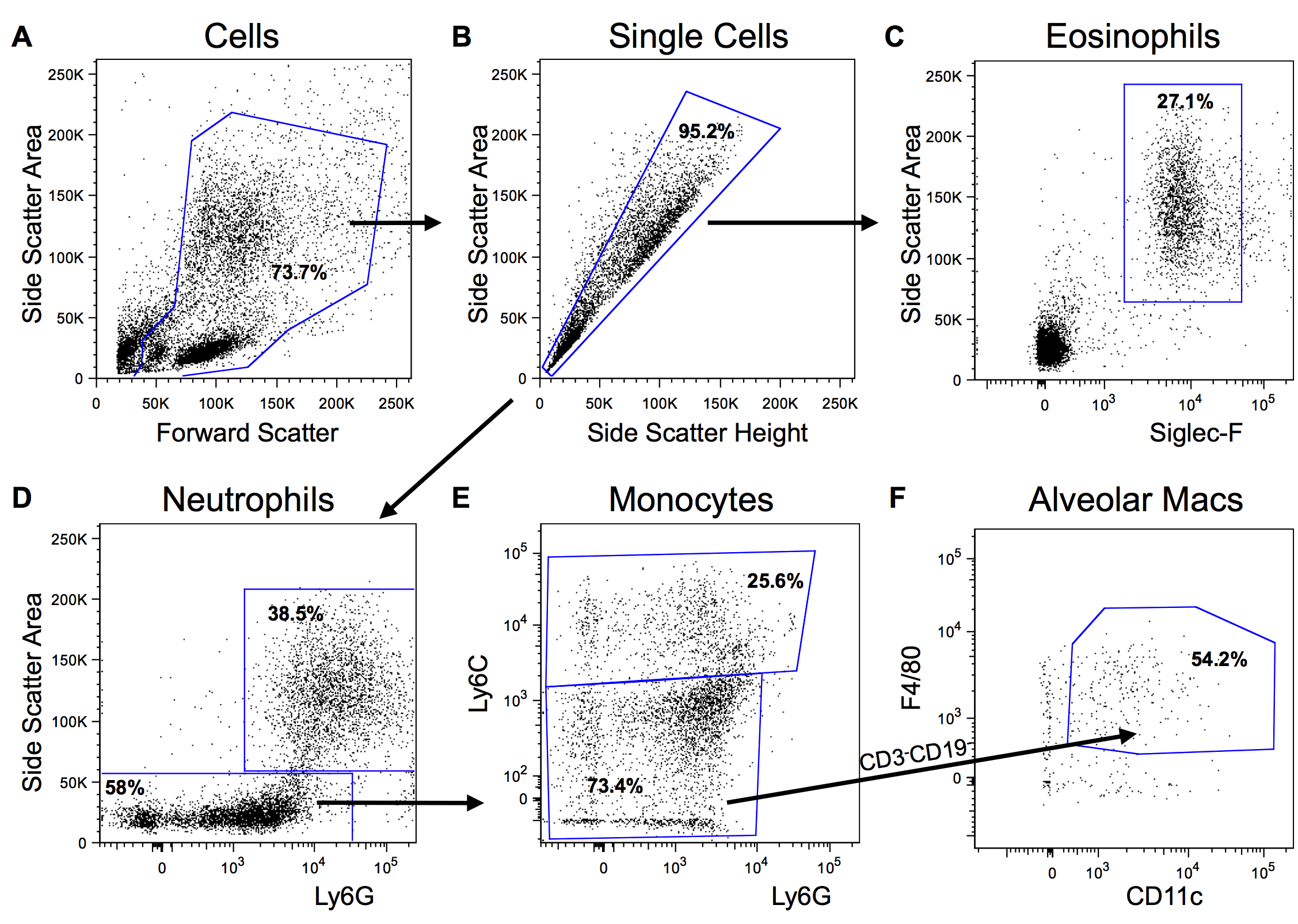

Supplement: Supplementary file 5 — Figure S5. Representative flow cytometric gating strategy for the CR extract model. For the flow cytometric phenotypic analysis, the cells of interest and exclusion of doublets were determined by the forward and side scatter parameters of the cellular populations (a, b). Siglec F was used to identify Eosinophils (c: SSChighSiglecF+), while a separate staining panel was used to identify Neutrophils (d: SSChighLy6G+). The Ly6G‐ population in D was further discriminated on the basis of their Ly6C expression (e) and following selection of the Ly6C‐Ly6G− CD3−CD19− cells, the subsequent use of F4/80 and CD11c allowed the identification of Alveolar Macrophages (f: Ly6C−Ly6G−CD3−CD19−CD11c+F4/80+). [file PIM-38-340-s005.tiff]
